# Alternative macrophage polarisation associated with resistance to anti-PD1 blockade is possibly supported by the splicing of FKBP51 immunophilin in melanoma patients

**DOI:** 10.1038/s41416-020-0840-8

**Published:** 2020-04-22

**Authors:** Teresa Troiani, Emilio Francesco Giunta, Martina Tufano, Vincenza Vigorito, Paolo D’ Arrigo, Giuseppe Argenziano, Fortunato Ciardiello, Maria Fiammetta Romano, Simona Romano

**Affiliations:** 1 Department of Precision Medicine, Oncology Unit, University of Campania Luigi Vanvitelli, Naples, Italy; 2 Department of Molecular Medicine and Medical Biotecnology, University of Naples Federico II, Naples, Italy; 3 Dermatology Unit, University of Campania Luigi Vanvitelli, Naples, Italy

**Keywords:** Melanoma, Prognostic markers

## Abstract

**Background:**

FKBP51 immunophilin is abundantly expressed by immune cells. Co-inhibitory immune receptor signalling generates the splicing isoform FKBP51s. Tregs stained by FKBP51s are increased in melanoma patients and their counts are associated with anti-CTLA-4 response. An expansion of FKBP51s^+^PD-L1^+^ monocytes was measured in a group of non-responding patients to anti-CTLA-4. The aim of this work was to confirm the predictive value of response of FKBP51s^+^Tregs in a cohort of patients undergoing anti-PD1 treatment and shed light on a monocyte subset co-expressing PD-L1/FKBP51s.

**Methods:**

Co-cultures of organoids and autologous lymphocytes were used to confirm that tumour T-cell interaction can induce FKBP51s. PBMC immunophenotype and flow cytometry served to assess and monitor FKBP51s^+^Treg and FKBP51s^+^PD-L1^+^ monocytes in 22 advanced melanoma patients treated with anti-PD1. Silencing and overexpression of FKBP51s in human macrophages served to address the protein role in the tolerant macrophages’ behaviour.

**Results:**

FKBP51s^+^Tregs count was increased in responders and had a prognostic value. Non-responders showed an early increase in FKBP51s^+^ PD-L1^+^ monocytes during anti-PD1 treatment. Manipulation of FKBP51s modulated the macrophage–phenotype, with forced protein expression promoting aspects associated with tolerance.

**Conclusions:**

FKBP51s may guide in the selection and monitoring of melanoma patient candidates to immune-checkpoint-targeted therapy. Manipulation of FKBP51s may overcome resistance.

## Background

The discovery of immune-checkpoint-targeted therapy has represented a breakthrough in advanced melanoma treatment. Approved medications include anti-PD1 (nivolumab and pembrolizumab), anti-CTLA-4 (ipilimumab) and anti-PD1/-CTLA-4 combination. Overall, these regimens work to induce reactivation of tumour-specific antigen-experienced T cells that destroy the tumour.^1,2^ Notwithstanding the dramatic outcomes of such an immunotherapeutic approach, high rates of clinically significant immune-related adverse events, especially with dual- combination regimens, occur that are often associated with no/poor response. Among mechanisms that contrast antitumour immune reactivation are lack of sufficient or suitable neoantigens, impaired neoantigen processing/presentation^1^ and recruitment of immune- suppressive cells (Tregs, MDSCs, Th2 CD4^+^ T cells and M2-polarised tumour-associated macrophages).^1–3^ So far, reliable response biomarkers are still lacking in clinical practice; notwithstanding efforts have been made in this regard. Studies have also been carried out on peripheral blood mononuclear cell immunophenotype to address deviations from healthy donor profiles that may uncover critical cell subsets associated with resistance/response.^4,5^ Distinct immune modulations that were connected to therapy response or tumour progression have been identified with PBMC immunophenotyping with prognostic and predictive relevance.^6^

Our group has recently identified a splice isoform of FK506-binding protein 51 (FKBP51, *FKBP5*) in PBMCs of melanoma patients, generated by an RNA-processing event induced by the co-inhibitory immune-checkpoint PD-L1/PD1 couple of ligand/receptor.^7^ FKBP51 is an immunophilin firstly cloned in lymphocytes^8^ and abundantly expressed by immune cells.^9^ This protein shares with other members of FKBP family proteins the domain of binding to the immunosuppressant agents FK506 and rapamycin. Such domain is endowed with an enzymatic function, namely peptidyl-prolyl-isomerase, catalysing the isomerisation of peptidyl bonds containing proline.^10^ In addition, FKBP51 has a three-tandem TPR repeat domain for protein–protein interaction.^11,12^ Thanks to its structure and function, FKBP51 participates as a scaffold and isomerase to IKK kinase complex assembly and activation, facilitating NF-κB induction by several stimuli.^13–16^ FKBP51s immunophenotyping of melanoma patients’ PBMCs showed that T-lymphocyte subsets and monocytes contained a proportion of an FKBP51s^+^ component, which resulted in significantly increased in melanoma patients.^17^ Intriguingly, the count of FKBP51s^+^Tregs resulted in a predictive response to ipilimumab, suggesting that this Treg subset develops in the context of an excess of co-inhibitory immune receptor signalling within the tumour microenvironment.^17^ In this view, we hypothesised that such a Treg subset could represent a guide in the selection of candidate patients to immunotherapy. Among checkpoint-targeted immunotherapies, neutralising antibodies targeting PD1 have shown to be particularly successful. According to long-term data, patients with melanoma treated with nivolumab, delivered either as monotherapy or in combination with ipilimumab, show superior OS, PFS and response rates compared with those on ipilimumab.^18^ In this work, we attempted to address our hypothesis of an association of the FKBP51s^+^Treg subset with the responder (R) profile to immune-checkpoint-targeted therapy in a cohort of patients undergoing anti-PD1 treatment. In addition, because our previous study found the expansion of an FKBP51s^+^PD-L1^+^CD14 monocyte population in a group of patients who were non-responding to ipilimumab,^17^ a further aim of this work was to shed light on such a CD14 subset. Alterations of the monocyte compartment in the peripheral blood during immunotherapy have been found by other authors.^3,5^ Several studies support the concept that tumour-associated macrophages protect cancer cells from the antitumour immune responses.^19,20^ By monitoring eventual changes occurring during the treatment, we aimed to address whether FKBP51s^+^PD-L1^+^CD14 subset can act as an early marker of resistance that informs on possible intervention changes. Our results show that FKBP51s^+^Tregs count is increased in R patients to anti-PD1, thus confirming its association with a condition that benefits from immune-checkpoint-targeted therapy. Moreover, we found that an increase in FKBP51s^+^PD-L1^+^CD14 monocytes occurred early during anti-PD1 treatment in non-responder (NR) patients. Such a monocyte subset shows high levels of *ARG1* and the macrophage scavenger receptor *MSR1* consistent with an alternative polarisation profile.

## Methods

### Patients and peripheral blood mononuclear cell (PBMC) isolation

PBMCs were isolated from the heparinised blood of 22 patients with advanced melanoma and age- and sex-matched healthy donors. More precisely, normal ranges were calculated on 64 and 27 healthy donors’ PBMCs, respectively, for Tregs and monocytes. Fresh tissue biopsies were obtained from two advanced melanoma patients undergoing anti-PD1 as first-line therapy. Blood and tissue samples were obtained from the Oncological Unit of the University of Campania “Luigi Vanvitelli”, as part of the routine management for patients with melanoma, following informed consent. The study was approved by the Ethics Committee of the University of Campania “Luigi Vanvitelli” (Protocol n° 59) and conducted in accordance with the ethical principles of the Declaration of Helsinki. Clinical information and the results of the study were handled by authorised personnel only. In compliance with patients’ rights, patient identity was kept confidential. Baseline demographical and clinical characteristics are listed in Table 1. Eleven patients were non-responders and 11 responders to anti-PD1 (nivolumab or pembrolizumab), according to iRECIST criteria.^21^ Between the two groups, age was well balanced (median: 69 vs 71 years; R vs NR), with a slight predominance of females in the R group (63.6%) and males in the NR group (54.5%). Concerning the stage of disease at baseline (according to AJCC staging system, VIII edition), half of NR patients (45.5%) had M1c disease, contrary to R patients, who had M1b as maximal tumour burden (18%). From the molecular point of view, R patients were all BRAF wild type and only one of them harboured a NRAS gene mutation; among NR patients, 3 (27.3%) had BRAF V600 mutant melanoma and 4 (36.4%) harboured NRAS mutations. All BRAF V600 mutant melanoma patients had received a combination of BRAF and MEK inhibitors before starting anti-PD1 treatment. PBMCs were isolated from 5 ml of heparinised blood collected in sterile K_3_EDTA vacutainer collection tubes. PBMCs were separated by differential centrifugation through a Ficoll-Hypaque density gradient (Histopaque-1077®, Sigma-Aldrich, St. Louis, MO, USA), washed and resuspended in 5% FBS-RPMI 1640 (Biowest, Nuaillè, France). After the count, PBMCs were processed for analysis by immunofluorescence.

**Table 1:** Patient characteristics.

	Responders (11)	Non-responders (11)
Age in years—median (range)	69 (41–84)	71 (31–82)
Sex (M:F)	1:1.75	1.25:1
Locally/advanced/inoperable	27.3% (3)	27.3% (3)
M1a	54.5% (6)	27.3% (3)
M1b	18.2% (2)	0% (0)
M1c	0% (0)	45.5% (5)
M1d	0% (0)	0% (0)
BRAF V600 mut	0% (0)	27.3% (3)
NRAS mut	18.2% (2)	36.4% (4)
Previous BRAFi + MEKi	0% (0)	27.3% (3)
PFS months—median (range)	17.3 (4.2–30.3)	3.1 (0.5–5.3)

### Generation of three-dimensional (3D) organoid cultures and co-culture with autologous lymphocytes

Fresh tissue biopsies derived from cutaneous lesions and metastasis at inguinal lymph nodes for patients A and B, respectively, were transported to the laboratory in order to establish 3D tumour organoid cultures. Tissues were weighed, washed twice with a 1× phosphate-buffered solution (PBS) and cut into fragments. Afterwards, tumour fragments were incubated with a digestion solution containing DMEM F-12 culture medium (Sigma-Aldrich), 2% Penicillin/Streptomycin (Biowest), 10× Amphotericin (Sigma-Aldrich), 2× Collagenase and Hyaluronidase (Sigma-Aldrich) for 6 h on a shaker at 37 °C in a 5% CO_2_-humidified atmosphere. All undigested fragments and debris were excluded by filtering through a cell strainer (Becton, Dickinson and Company BD, Franklin Lakes, NJ, USA) and the filtered and digested solution was then subjected to centrifugation for 5 min at 1200 rpm. The supernatant was removed, and the pellet was washed with 1× PBS and resuspended in an ice-cold 1:1 mixture of growth medium and matrigel (Becton, Dickinson and Company BD) and then seeded in ultra-low-attachment 24-well plates (Corning, NY, USA). The matrigel droplets were left to polymerise for 10 min at 37 °C and growth medium was added after polymerisation. Morphologies of 3D tumour organoids were followed day by day, and images were captured by a microscope using a ×10 objective. For co-culture experiments, autologous PBMCs were thawed, and lymphocytes were isolated from monocytes by plate adherence for 6 h. Organoids and lymphocytes were seeded in matrigel, plated in duplicate as droplets in the centre of 24 ultra-low-attachment plates. After 24 h of co-cultures, matrigel was degraded using the Cell Recovery Solution (Becton, Dickinson and Company BD) according to the manufacturer’s procedures, which enabled cell suspension collection by centrifugation for 5 min at 1200 rpm. Cells were then subjected to immunofluorescence to analyse PD-L1 and FKBP51s expression in lymphocytes (CD45^+^/CD3^+^) and organoids (CD45^-^); further details for sample staining and analysis are described in the “Flow cytometry analysis” section.

### Confocal microscopy

For immunofluorescent staining of S100 and MART-1 markers, 3D tumour organoids were embedded in matrigel and seeded on a chamber slide (Thermo Fisher Scientific, Waltham, MA, USA). 3D organoids were fixed with 4% paraformaldehyde for 30 min and permeabilised with 0.1% Triton X-100 (Sigma-Aldrich) before incubation with rabbit anti-S100 (Dako Agilent Technologies, Santa Clara, CA, USA) and mouse anti-MART-1 (Dako Agilent Technologies) primary antibody. Afterwards, Alexa Fluor488 goat anti-rabbit secondary antibody (Invitrogen, Carlsbad, CA, USA) was used. Cell nuclei were stained with 4.6-diamidino-2-phenylindole (DAPI, Sigma-Aldrich). Images were captured with an inverted Zeiss LSM 700 microscope, using a ×20 objective (Carl Zeiss, Oberkochen, Germany); images were acquired with the Zeiss software with a 1024 × 1024-pixel resolution, a thickness of each z slice was set to 1.6 μm and were shown as the maximum projection of each z slide acquired. When required, the brightness, contrast and colour balance of the images were adjusted in Photoshop CS2 (Adobe Systems, San Jose, CA, USA). This adjustment was applied to every pixel in each image.

### Co-culture suppression assays

Human PBMC-derived macrophages were prepared by plate adherence and transfected as described in the “Cell transfection” section. Non-adherent cells were processed through the MACS® Technology using human CD4 MicroBeads (Miltenyi Biotec, Bergisch Gladbach, Germany) for the positive selection of CD4^+^ cells by direct magnetic labelling. CD4^+^ cells were assessed for their purity and viability and used for the suppression assay. Briefly, part of CD4^+^ cells was stained with the carboxyfluorescein succinimidyl ester (CFSE) (Thermo Fisher Scientific), according to the manufacturer’s instructions, before co-culture. The cell suspension was washed two times with PBS to remove any serum and resuspended at the concentration of 5 × 10^6^ cells/mL of pre-warmed PBS. One μM CFSE/mL of cells was used for labelling; cells were immediately mixed and incubated for 10 min at room temperature in the dark. Labelling was stopped by adding 4–5 volumes of cold complete 10% FBS-culture medium and incubated on ice for 5 min. Cells were then washed three times with 10% FBS-culture medium and employed for the suppression assay. To this end, 25 × 10^3^/well macrophages and 10–20 × 10^4^ CD4 lymphocytes (CFSE-stained or not) were co-cultured in a total volume of 200 μl of 10% FBS-culture medium with CD3 and CD28 Monoclonal Antibodies, Functional Grade, eBioscience™ (Thermo Fisher Scientific), and used 0.25 and 1 µg per 10^6^ cells. CD4 lymphocytes were harvested from 1 to 6 days; those stained with CFSE were labelled with propidium iodide for 10 min in the dark in order to easily identify viable cells. CD4 cells, not labelled with CFSE, were stained to measure Ki67 expression (see “Flow cytometry analysis” section for further details). The analysis was performed by flow cytometry.

### Flow cytometry analysis

BD-Pharmigen Fc block (2.5 μg/10^6^ cells) was used to minimise non-specific binding of immunoglobulins to Fc receptors, prior to the immunostaining. PBMCs were subjected to a multiple-immunofluorescence staining. For this purpose, 5–10 μl (in accordance with concentration and the manufacturer’s instruction) of mouse monoclonal antibody recognising the typical cluster differentiation (CD) was added to 50 μl of PBMC suspension. Cells were incubated for 15 min in the dark at room temperature (20–25 °C). The following antibodies were used: anti-CD4-PerCP (VIT4 clone, Miltenyi Biotec), anti-CD14-PerCP (TÜK4 clone, Miltenyi Biotec); anti-PD-L1-Phycoerythrin (PE) (MIH1 clone, eBioscience), anti-CD80-APC (MEM233, Immunotools) and anti-HLA-DR-PE (L243 clone, Invitrogen). Next, 200 μl of a fixation/permeabilisation buffer (BD-Pharmingen Cytofix/CytopermTM Kit, San Jose, CA, USA) was added to each tube and incubated for 20 min in the dark at 4 °C. After fixation and permeabilisation, the cells were further incubated for intracytoplasmic staining by direct immunofluorescence with anti-FKBP51s using anti-FKBP51s antibody conjugated with the 5-carboxyfluorescein (FAM) as previously described^17^ and the anti-IL-10-PE (JES3-9D7 clone, eBioscience™, Thermo Fisher Scientific). To make the nuclei accessible, before staining with an anti-Foxp3-PE antibody (PCH101 clone, eBioscience™, Thermo Fisher Scientific) and anti-Ki67 (REA183 clone, Miltenyi Biotec), cell fixation and permeabilisation were performed using the Foxp3/Transcription Factor Staining Buffer Set (eBioscience™, Thermo Fisher Scientific). For each staining, a relative Ig isotype-conjugated antibody was used as a control of non-specific binding. Lymphocyte and monocyte gating and subset counts were performed as previously described.^17^ Samples were analysed using a BD Accuri™ C6 Cytometer (Becton, Dickinson and Company BD). The flow cytometry data were analysed by using the FlowJo software or the C6 Accurì software.

### qPCR

Total RNA extraction was performed using TRIzol (Sigma-Aldrich), according to the manufacturer’s instructions. One μg of each RNA was used for cDNA synthesis with iScript™ Reverse Transcription (Bio-Rad, Hercules, CA, USA). Gene expression was quantified by quantitative (q) PCR using SsoAdvanced™ SYBR Green Supermix (Bio-Rad) and specific qPCR primers for the relative quantitation of the transcripts, performed using co-amplified β-Actin^16^ as an internal control for normalisation. Oligo sequences are reported: h-ARG1-Fw: 5′-GGCTGGTCTGCTTGAGAAAC-3′; h-ARG1-Rev: 5′-CTTTTCCCACAGACCTTGGA-3′; h-MSR1-Fw: 5′-CCTCGTGTTTGCAGTTCTCA-3′; h-MSR1-Rev: 5′-CCATGTTGCTCATGTGTTCC-3′. Transcript levels of FKBP51s were measured as described.^7^

### Cell transfection

Human PBMCs isolated from peripheral blood of healthy donors were cultured in RPMI 1640 medium with 2 mM L-glutamine (Corning) and without serum in 15-ml polypropylene tubes to be further processed for flow cytometric experiments. Human PBMC-derived macrophages were prepared by plate adherence^22^ for 4 h, washed with fresh medium to remove unattached cells and incubated overnight to let them differentiate and proliferate sufficiently to become confluent and be used for experiments. Cell transfection was performed using a K2 Transfection System (Biontex, Munich, Germany), according to the manufacturer’s instructions. For knockdown experiments, cells were transfected with specific short-interfering oligoribonucleotide (siRNA) (or with a non-silencing oligoribonucleotide, NS RNA, as control) at a final concentration of 50 nM. NS RNA was purchased from Novus Biological (Littleton, CO, USA). For FKBP51s siRNA, a mixture of three different siRNAs was used as previously described.^23^ To overexpress FKBP51s, a True-ORF-Myc-DDK-tagged expression vector was used (OriGene Technologies, Rockville, MD, USA). This vector carried the cDNA of the human *FKBP5* transcript variant 4. The relative void vector (EV) was also transfected to generate control cells. About 16 h after transfection, cells were collected or detached to be further processed.

### Immunoblot

Whole-cell lysates were homogenised in modified RIPA buffer^15^ and assayed by immunoblot. The primary antibody against FKBP51s,^7^ phospho-STAT3 (Tyr 705, rabbit polyclonal, GeneTex, Irvine, CA, USA), STAT3 (mouse monoclonal, Cell Signaling, Danvers, MA, USA) and Vinculin (mouse monoclonal, Santa Cruz Biotechnology, Dallas, Texas, USA) was used and diluted 1:2500, 1:3000, 1:1000 and 1:5000, respectively. Protein samples were then separated by SDS-PAGE.

### Statistical analysis

Student’s *t* test was used to analyse the differences between the means of values; *p* value ≤ 0.05 was considered statistically significant. The receiver-operating characteristic (ROC) curve was used to establish sensitivity, specificity and predictive values of FKBP51s Tregs count according to the response to anti-PD1 therapy (R vs NR). The progression-free survival (PFS) rate was estimated by Kaplan–Meier method to generate survival curves, and the significance of the difference between survival curves was calculated by the log-rank test (Mantel–Cox).

## Results

### Organoids generated from melanoma tumours can affect FKBP51s expression in co-cultured autologous PBMCs

**Fig. 1:**
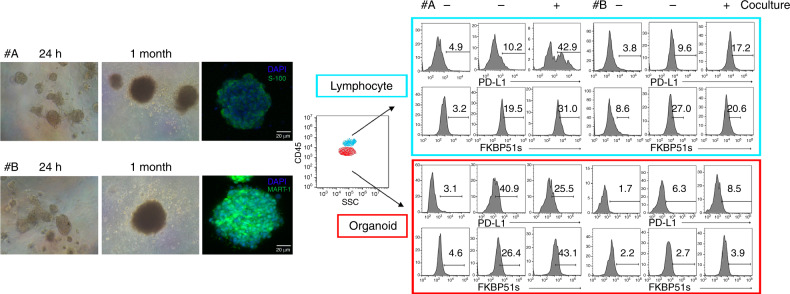
Organoids generated from melanoma tumours can affect FKBP51s expression in co-cultured autologous PBMCs. On the left, organoids captured by optical microscope after 24 h and 1 month of cell growth in matrigel, and by fluorescent microscopy after staining with s100 (#A) or MART-1 (#B). On the right, flow cytometry histogram of PD-L1 and FKBP51s expression in organoids (CD45^−^ cells, red square) and T lymphocytes (CD45^+^ CD3^+^, light-blue square). PD-L1 expression was 40.9% and 6.3%, respectively, in organoids #A and #B. FKBP51s levels were increased in co-cultures of patient #A.

We have previously shown that tumour-immune cell interaction through PD-L1/PD1 bidirectionally stimulated *FKBP5* alternative splicing.^7,17^ We have also shown that expression of FKBP51s in tumour-infiltrating lymphocytes of melanoma tissues is influenced by tumour PD-L1 expression.^7^ Herein, using organoids generated by two different melanoma tissue samples and autologous PBMC co-cultures, we provide further evidence of the causative effect of PD-L1/PD1 interaction on *FKBP5* alternative splicing. Figure 1 shows that organoids produced by two metastases (patient #A and patient #B) were efficiently generated. The proportion of CD3+ T lymphocytes was 73.1% and 95.2% in PBMCs from patients #A and #B, respectively. Patient #A but not #B responded to nivolumab as first line of therapy. Interestingly, we measured 40.9% of PD-L1 expression in organoid #A and only 6.3% in #B by flow cytometry (Fig. 1). In organoid #A, we registered a decrease in PD-L1 after co-culture from 40.9% to 25.5%. After co-culture, an increase in FKBP51s was measured in both organoid #A and in autologous CD3 T lymphocytes. In organoid #A, the proportion of FKBP51s expression increased from 26.4% (basal value) to 43.1% (value in co-culture). In CD3 T-lymphocyte #A, FKBP51s increased from 19.5% (MFI 2584, basal value) to 31.0% (MFI 4194, value in co-culture). Levels of FKBP51s in organoid #B appeared to be low (2.7% and 3.9%, basal and after co-culture). Organoid #B did not produce in CD3 T lymphocytes any increase in FKBP51s (basal value: 27.0%, MFI 3003, and co-culture value: 20.6%, MFI 2838). Expression of PD-L1 in co-cultured lymphocytes appeared to be particularly increased in #A, from 10.2% to 42.9% and slightly increased in #B, from 9.6% (basal level) to 17.2% (value of co-culture). These results support previous findings that FKBP51s is a molecular sensor of a co-inhibitory immune receptor signaling.^7,17^ The different lymphocyte outcomes in organoid co-cultures can be explained in view of a higher PD-L1 expression on organoid #A with respect to organoid #B, which reasonably favours a major interaction between tumour and lymphocytes, in accordance with Kluger et al.^24^ On co-cultured organoid #A with lymphocytes, PD-L1 downregulation may be a consequence of such an interaction, given that PD-L1 internalisation transiently occurs after ligand binding.^25^ Increased PD-L1 level in activated lymphocytes is induced by multiple pro-inflammatory molecules, with IFN-γ being the most potent inducer.^26^ At the same time, the levels of its cochaperone FKBP51s increase as a consequence of the alternative splicing of FKBP5 generated by PD-L1-PD1 interaction,^7^ to support PD-L1 glycosylation and expression on plasma membrane.^23^

### Increased counts of FKBP51s+Tregs in responder patients to anti-PD1

**Fig. 2:**
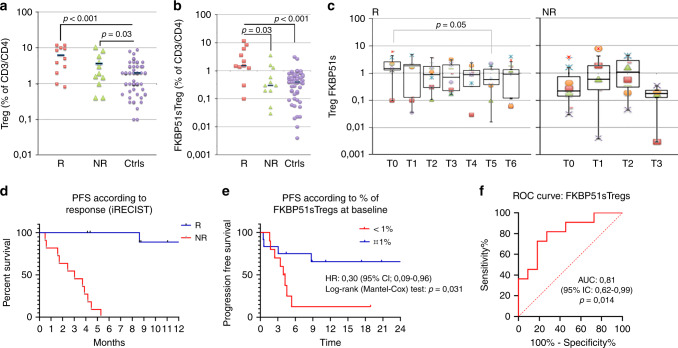
Increased counts of FKBP51s^+^ Tregs in responder patients to anti-PD1. **a** Total Treg counts and **b** FKBP51s Treg counts in cases (11, R, and 11, NR) and controls (*n* = 64). **c** Graphic representation of FKBP51s Treg counts at baseline and during treatments from 8 R and 8 NR patients. **d** PFS curves in R and NR patients: median PFS was 2.8 months in NR and not reached in R. **e** PFS curves according to FKBP51s Treg values, using a threshold of 1%: median PFS was 4.2 months and not reached in patients with <1% or ≥1% of FKBP51s Tregs, respectively. **f** ROC curve according to FKBP51s Treg values.

FKBP51s Tregs have been firstly identified in melanoma patients.^17^ In particular, the patients who were responding to ipilimumab showed an increased count of this T-cell subset.^17^ We examined the level of total Tregs and FKBP51s Tregs in 22 patients receiving anti-PD1 treatment (nivolumab or pembrolizumab) (see Table 1 for patient profile). The response rates for anti-PD1 (11/22, 50%) were higher than those previously found with anti-CTLA-4 (18/64, 28%).^17^ Figure 2a, b shows the total Treg and FKBP51s Treg counts, as assessed before anti-PD1. We measured an increased level of Tregs in melanoma patients compared with age-matched healthy donors (controls) (Fig. 2a). However, FKBP51s Tregs were significantly increased only in R patients, compared with NR and controls, these two latter showed comparable levels (Fig. 2b). FKBP51s Tregs from 8 R patients and 8 NR were monitored at each bi- or triweekly treatment. R was monitored from T0 (baseline) to T6 (after 2–3 weeks from the 6th treatment); NR from T0 to T3 (after 2–3 weeks from the 3rd treatment) because of reduced cooperative behaviour (Fig. 2c). A significant downmodulation of FKBP51s Tregs was registered at T5 in R. No significant modulation of FKBP51s Tregs was registered during the treatments in NR. Also, no significant modulation of total Tregs was registered during the treatments, in both R and NR (see supplementary information Fig S1). These findings suggest that anti-PD1 can lately affect the count of FKBP51s Tregs but total Tregs. Moreover, in the overall population, the median PFS was 3.1 months (95% IC: 0.5–5.3) in NR and not reached in R (Fig. 2d). However, to better stratify patients, PFS curves were obtained according to FKBP51s Treg values at baseline, using a threshold of 1% value, as it was previously calculated.^17^ Median PFS in patients with percentage <1% was 4.1 months (1.6 month—not reached), whilst in patients with percentages, ≥1% was not reached (0.5 month—not reached), with a hazard ratio of 0.30 (0.09–0.96, *p* = 0.03) (Fig. 2e). Sensitivity, specificity and predictive values of baseline FKBP51s Treg values have also been calculated with the same threshold of 1%, using the receiver-operating characteristic (Fig. 2f). AUC was 0.81 (95% IC: 0.62–0.99, *p* = 0.014). We obtained a sensitivity of 81.8% and a specificity of 72.7%, with a positive predictive value (PPV) of 75% and a negative predictive value (NPV) of 80%.

### Increased counts of FKBP51s^+^ PD-L1^+^ monocytes in non-responder patients to anti-PD1

**Fig. 3:**
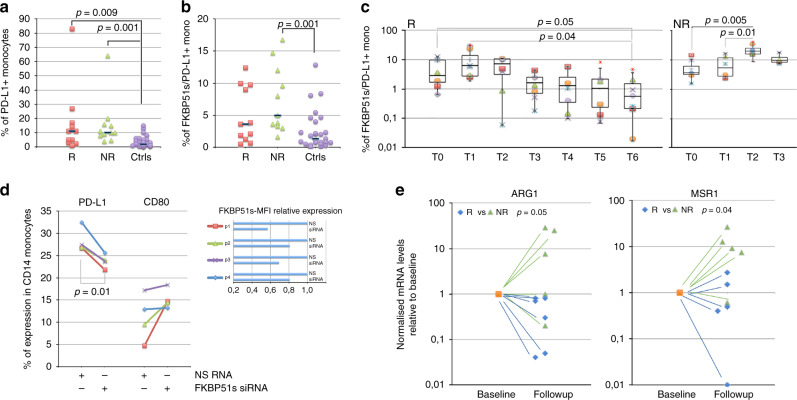
Increased counts of FKBP51s^+^ PD-L1^+^ monocytes in non-responder patients to anti-PD1. **a** Total PD-L1^+^ monocyte counts and **b** FKBP51s^+^ PD-L1^+^ monocyte counts in cases (11, R, and 11, NR) and controls (*n* = 27). **c** Graphic representation of FKBP51s^+^ PD-L1^+^ monocyte counts at baseline and during treatments from 8 R and 8 NR patients. **d** Effect of FKBP51s downmodulation on PD-L1 and CD80 expression from four patients. Expression values were assessed in flow cytometry, in the absence (NS RNA) or the presence (FKBP51s siRNA) of silencing. On the right, the changes in mean fluorescence intensities (MFI) of FKBP51s are also represented. **e** Levels of *ARG1* and *MSR1* in 6 R and 5 NR patients were assessed by qPCR at baseline and at T2/T3 (follow-up). Values at follow-up were expressed as fold change, using the respective baseline value as reference sample (=1).

Several studies support the concept that tumour-associated macrophages protect cancer cells from the antitumour immune responses.^19,20^ Because we have previously found that a monocyte subset co-expressing PD-L1 and FKBP51s appeared to be associated with resistance to ipilimumab, we measured the counts of FKBP51s^+^PD-L1^+^ CD14 mononuclear cells in our study population, at baseline and during the treatments. As shown in Fig. 3a, the baseline count of PD-L1^+^ monocytes was significantly increased in both R and NR, with respect to the controls. Differently, FKBP51s^+^ PD-L1^+^ monocytes were significantly increased only in NR, with respect to the controls (Fig. 3a). Specifically, the count of FKBP51s^+^ PD-L1^+^ monocytes ranged from 0.7% to 5.2% (median 3.1%) in controls, from 1.5% to 9.3% (median 3.6%) in R and from 3.3% to 9.2% (median 4.4%) in NR (*p* = 0.03 vs controls) (Fig. 3b). By monitoring the FKBP51s^+^ PD-L1^+^ monocyte subset during the treatment, we found that in NR patients, contrarily to what occurred for R patients, the counts further increased at T2, with respect to baseline level (Fig. 3c). Interestingly, the silencing of FKBP51s of PBMCs from four patients produced a significant downmodulation of PD-L1 accompanied by an increase, albeit not significant, of CD80 (Fig. 3d). Because tumour-associated macrophages display shifted arginine metabolism towards the production of ornithine and polyamines via arginase^27^ along with high levels of scavenger receptors,^28^ we thought to measure by qPCR, whether the levels of *ARG1* and the macrophage scavenger receptor *MSR1* in the study population changed during the treatment (baseline vs T2/T3). As shown in Fig. 3e, levels of *ARG1* and *MSR1* increased during treatment, while those measured in R patients decreased.

### FKBP51s can promote alternative monocyte polarisation

**Fig. 4:**
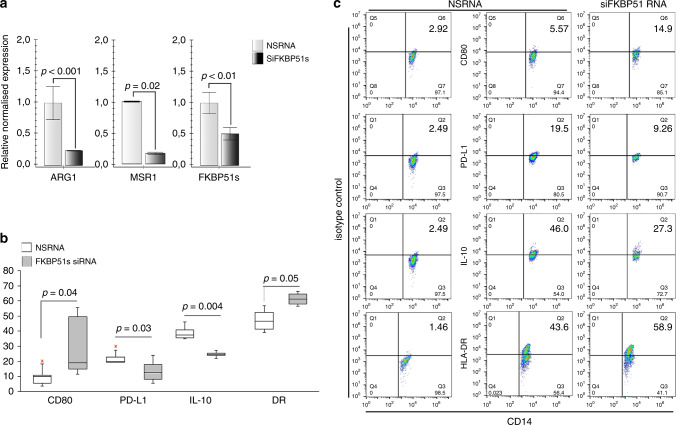
Effect of FKBP51s downregulation on expression of monocyte markers associated with positive (HLA-DR, CD80) or negative (PD-L1, IL-10, ARG1 and MSR1) lymphocyte co-stimulation. **a** QPCR measure of *ARG1* and *MSR1* transcript levels in RNA extracted by healthy donors’ macrophages downmodulated or not for FKBP51s. Values of FKBP51s-silenced monocytes were expressed as fold change, using the non-silenced monocyte value as reference sample (=1). **b** Graphic representation of flow cytometric values of expression of CD80, PD-L1, IL-10 and HLA-DR in macrophages downmodulated or not for FKBP51s, from four healthy donors, and **c** representative flow cytometry histograms.

**Fig. 5:**
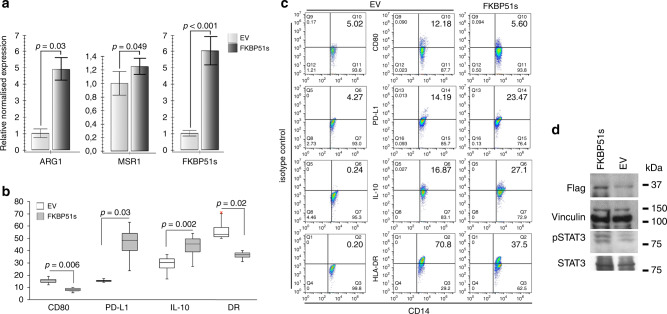
Effect of forced FKBP51s expression on monocytes’ co-stimulation markers. **a** QPCR measure of *ARG1* and *MSR1* transcript levels in RNA extracted by healthy donors’ macrophages transfected with FKBP51s or empty vector (EV) as control. Values of FKBP51s- overexpressing monocytes were expressed as fold change, using the EV-monocyte value as reference sample (=1). **b** Graphic representation of flow cytometric values of expression of CD80, PD-L1, IL-10 and HLA-DR, in EV- or FKBP51s macrophages, from four healthy donors, and **c** representative flow cytometry histograms. **d** Western blot assay of pSTAT3 levels in FKBP51s macrophages.

**Fig. 6:**
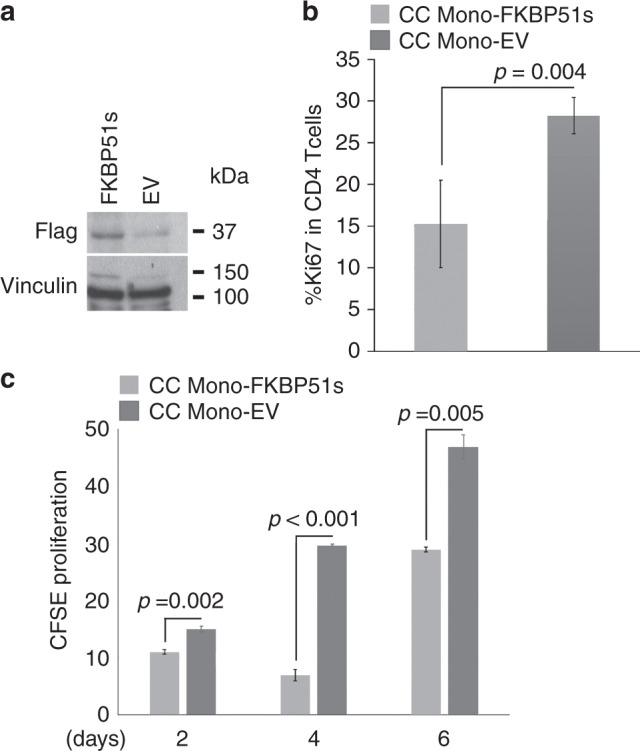
Inhibitory effect of FKBP51s monocytes on lymphocyte proliferation. **a** Overexpression of FKBP51s in human macrophages was assessed by western blot. **b** Graphic representation of Ki67 expression levels, as assessed by flow cytometry, on lymphocytes co-cultured with FKBP51s- or EV macrophages (*N* = 3). **c** Graphic representation of proliferation, as assessed by CFSE and flow cytometry, of lymphocytes co-cultured with FKBP51s- or EV macrophages (*N* = 3).

Because monocytes from NR patients showed aspects of M2 polarisation, and as FKBP51s appeared to oppositely regulate expression of the co-inhibitory immune-checkpoint PD-L1 and the co-stimulatory CD80, we downmodulated FKBP51s in monocytes isolated by peripheral blood of three healthy donors, through plate adherence, and evaluated the expression levels of *ARG1* and *MSR1* by qPCR. In addition, we measured by flow cytometry the expression of co-stimulatory antigens as MHC class II and CD80 along with inhibitory factors as the cytokine IL-10 and PD-L1 in monocytes from four healthy donors. Figure 4a shows that the downmodulation of FKBP51s was accompanied by a decrease in *ARG1* and *MSR1*. Expression of CD80 and HLA-DR resulted in an increase, whereas IL-10 and PD-L1 decreased by FKBP51s silencing (Fig. 4b, c). To reinforce the role of FKBP51s in promoting the alternative monocyte polarisation, we overexpressed this protein in monocytes from the peripheral blood of four healthy donors. As shown in Fig. 5a, the levels of *ARG1* and *MSR1* resulted in an increase in FKBP51s monocytes in comparison with monocytes transfected with the empty vector (EV). Expression of CD80 and HLA-DR resulted in a decrease, whereas IL-10 and PD-L1 increased by FKBP51s overexpression (Fig. 5b, c). Moreover, in accordance with the notion that IL-10 promotes STAT3 activation, we found increased levels of pSTAT3 in FKBP51s-overexpressing monocytes (Fig. 5d). Taken together, these findings suggest a role for FKBP51s in promoting M2 polarisation. To further reinforce this hypothesis, we evaluated the ability of FKBP51s-overexpressing monocytes to induce immune tolerance. To this end, we co-cultured FKBP51s or EV monocytes with CD3-stimulated purified CD4 T lymphocytes and measured proliferation by CFSE and Ki67 expression. The results from three donors are represented in Fig. 6. A proliferation-inhibitory effect by FKBP51s monocytes was measured, which suggests that the splicing protein effectively promotes a tolerant monocyte–macrophage behaviour.

## Discussion

FKBP51s has been recently identified in the peripheral blood of melanoma patients, and little is known on its function. With respect to FKBP51, FKBP51s lacks the TPR domain that is essential for the IKK kinase complex assembly.^16^ Therefore, reasonably, the occurrence of this splice isoform downstream to a co-inhibitory immune receptor signalling may possibly concur to dampen lymphocyte activation by attenuating NF-κB transcriptional activity. This hypothesis deserves to be addressed in the future. In glioma cell lines, co-immunoprecipitation assays of proteins extracted by endoplasmic reticulum showed an interaction between FKBP51s and PD-L1.^22^ Moreover, FKBP51s isomerase activity was found essential to assist in glycosylation and expression on the plasma membrane of this immune-inhibitory molecule.^22^ Although few, these evidences suggest that FKBP51s can be better suited than FKBP51 to an immune-tolerant phenotype. FKBP51s marks a subset of Tregs, which, according to increased p-mTOR levels,^17^ is expected to exert an effective suppressive activity.^29^ Herein, investigating a different cohort of melanoma patients, we confirm previous findings^17^ that this Treg subset is associated with response to checkpoint-targeted therapy. In addition, we show that the expansion of FKBP51s Tregs is a favourable prognostic factor in patients undergoing anti-PD1 treatment. Interestingly, this result is in line with a study by Correale et al. who found that a higher Foxp3^+^ T-lymphocyte tumour infiltration score is a favourable prognostic factor in colon cancer patients undergoing chemo- or chemoimmunotherapy.^30^ In accordance with other studies that report no change in the frequency of Treg cells in response to nivolumab or pembrolizumab, as reviewed in ref., ^31^ we found that the counts of FKBP51s Tregs were not significantly affected by anti-PD1, if not belatedly after at least 10 weeks from starting treatment. The precise effect of PD1 blockade on Treg cells is poorly understood. It is important to note that PD1 and PD-L1 are expressed by Treg cells; thus, direct modulation of Treg cell function should not be excluded as a possibility.^31^ Our results reinforce the concept that FKBP51s Tregs count at baseline can be a valuable tool to select candidate patients to immunotherapy but, at the same time, it cannot be a suitable parameter to monitor patients’ response to immune-checkpoint inhibitors. Our study points to a monocyte subset FKBP51s^+^ PD-L1^+^ as a valuable element for immunotherapy monitoring. We found, indeed, that this CD14 subpopulation significantly increased as soon as after 4 weeks from treatment starting in NR patients. The observation that the treatment produced an increase in the expression levels of *ARG1* and *MSR1* in NR, while a decrease in R patients supported the hypothesis that an alternative polarisation of macrophages occurred, improves tumour tolerance and generates anti-PD1 resistance. Ex vivo experiments with patients’ monocytes showed that, upon FKBP51s silencing, PD-L1 expression resulted in downmodulation, suggesting that *FKBP5* splicing supported changes in monocyte function. Such a hypothesis was confirmed by experiments of FKBP51s silencing and overexpression in primary monocytes from healthy donors. FKBP51s silencing produced, indeed, a decrease in expression levels of *ARG1* and *MSR1* mRNAs, PD-L1 on the plasma membrane and intracellular IL-10. In the same cells, an increase in CD80 and HLA-DR expression levels was registered. The opposite occurred with FKBP51s overexpression, which reinforced downmodulation data. In addition, we also show that, upon FKBP51s overexpression, the levels of pSTAT3 resulted in increased, in accordance with the IL-10 upregulation. The tolerant profile of FKBP51s-overexpressing CD14 cell was assessed in co-culture assays with CD3-stimulated lymphocytes, showing that, in comparison with macrophages transfected with the empty vector, those overexpressing FKBP51s have a reduced efficacy to act as accessory cells capable of co-stimulating lymphocyte proliferation. In conclusion, our study proposes that *FKBP5* splicing generated by co-inhibitory immune receptor signalling sustains a tolerant phenotype of immune cells. While the activity of FKBP51s Tregs appears to be effectively contrasted by anti-PD1, FKBP5 splicing in tumour-associated macrophages favours tumour tolerance and anti-PD1 resistance. More studies are needed to understand the reasons for the development of such immune-suppressive macrophages during anti-PD1 treatment. Predictive and prognostic markers that are easily accessible biomarkers for response to immunotherapy are urgently needed. Even if a note of caution must be given due to the low number of patients enrolled, our results suggest increased numbers of FKBP51s Tregs and lower numbers of FKBP51s^+^/PD-L1^+^ monocytes as favourable prognostic markers. Moreover, this explorative study suggests that manipulating *FKBP5* splicing is a promising strategy for targeting tumour-associated macrophages and reprogramming these cells from immune suppressive to immune-activating and tumouricidal ones.

## Supplementary information

Supplemental material

## Data Availability

The authors confirm that the data supporting the findings of this study are available within the article and its Supplementary Materials.
