# Supplementary material for: Alternative macrophage polarisation associated with resistance to anti-PD1 blockade is possibly supported by the splicing of FKBP51 immunophilin in melanoma patients
Source: Br J Cancer. 2020 Apr 22;122(12):1782–90. doi: 10.1038/s41416-020-0840-8 (PMC7283486; doi:10.1038/s41416-020-0840-8)
Supplement: Supplementary file 1 — Supplemental material [file 41416_2020_840_MOESM1_ESM.docx]

Supplementary information


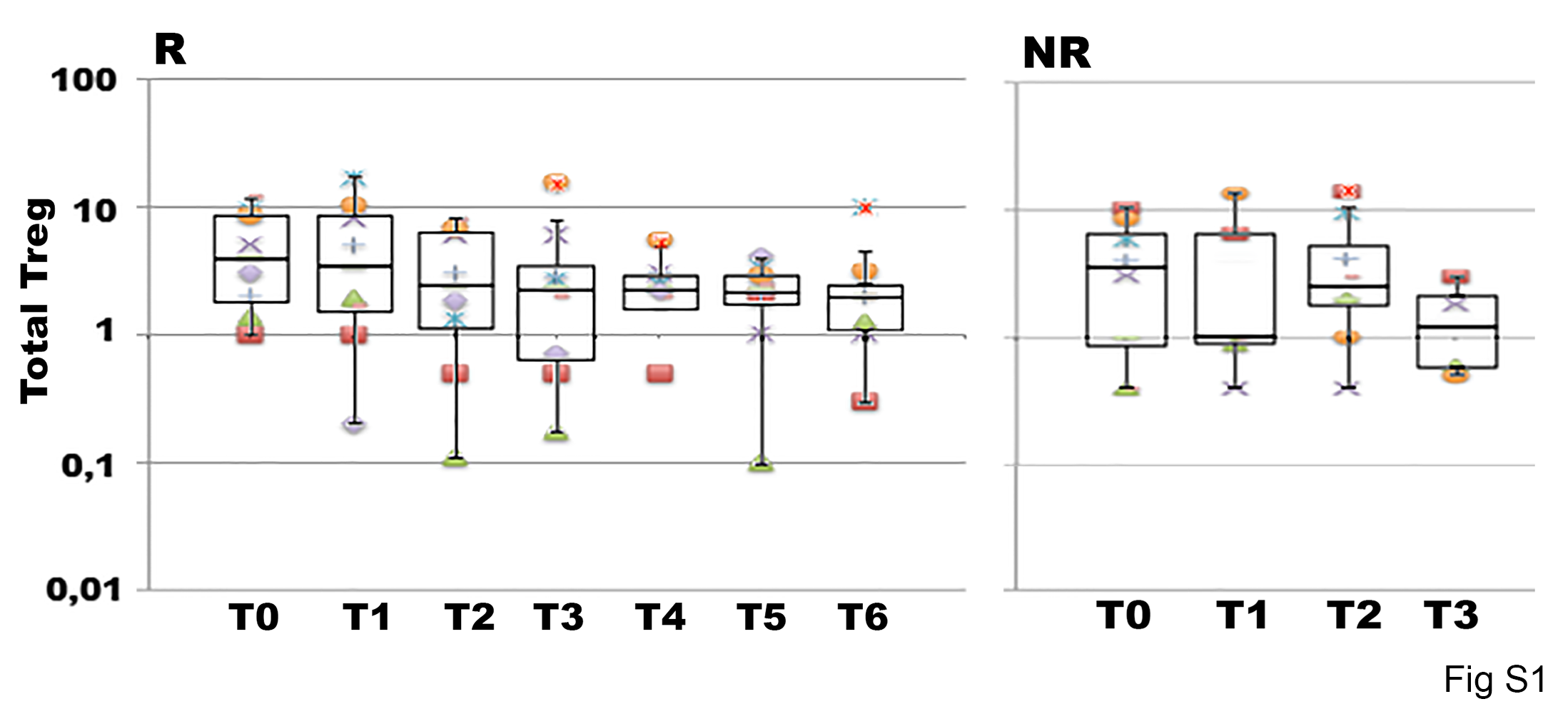


Fig S1 Graphic representation of Tregs counts at baseline and during treatments from 8 R and 8 NR patients.
